# Exploration or exploitation? The double-edged effects of AI use modes on radical creativity: evidence from AI-enabled drug discovery

**DOI:** 10.3389/fpsyg.2026.1931423

**Published:** 2026-10-13

**Authors:** Haoyu Wang, Zhi Xu, Zhihong Huang, Huaihong Pan, Guanbin Zheng

**Affiliations:** School of Business Administration, South China University of Technology, Guangzhou, China

**Keywords:** AI-enabled drug discovery, environmental dynamism, exploitative AI use, exploratory AI use, radical creativity, temporal myopia

## Abstract

Artificial intelligence (AI) has been widely adopted in corporate innovation activities, yet prior research has not reached a consensus on whether it is more conducive to incremental or radical innovation. For radical innovation, which depends on highly novel ideas, realizing the value of AI depends not only on whether AI is adopted but, more importantly, on whether employees can use it creatively to transcend established knowledge boundaries. Drawing on environmental dynamism and the exploration–exploitation perspective, this study develops a theoretical model linking leaders’ perceptions of environmental dynamism, employees’ AI use modes, leader temporal myopia, and employee radical creativity in the context of AI-enabled drug discovery. We distinguish leaders’ perceptions of environmental dynamism into perceived technological dynamism and perceived market dynamism, and employees’ AI use into exploratory and exploitative AI use. The model is tested using partial least squares structural equation modeling and three-wave matched survey data from 368 leader–employee dyads. The results show that leader-perceived technological dynamism is positively associated with employee exploratory AI use, whereas leader-perceived market dynamism is positively associated with employee exploitative AI use. Exploratory AI use is positively associated with employee radical creativity, whereas exploitative AI use is negatively associated with it. Leader temporal myopia weakens the positive conditional indirect relationship of technological dynamism with radical creativity through exploratory AI use and strengthens the negative conditional indirect relationship of market dynamism with radical creativity through exploitative AI use. By adopting a knowledge-activity perspective, this study reveals the differentiated relationships between AI use modes and radical creativity and further explains how perceptions of the external environment, leaders’ temporal orientation, and employees’ AI use behaviors are differentially related. The findings provide a theoretical basis and practical implications for helping firms engaged in AI-enabled drug discovery guide employees toward effective AI use and enhance radical creativity.

## 1 Introduction

As artificial intelligence (AI) becomes increasingly embedded in target identification, molecular generation, virtual screening, property prediction, and preclinical development, AI-enabled drug discovery has been widely regarded as an important technological pathway for improving R&D efficiency and expanding the potential for radical innovation (Sadybekov and Katritch, 2023; Vamathevan et al., 2019). However, the integration of AI does not automatically translate into radical innovation. Current practices in AI-enabled drug discovery remain largely shaped by an emphasis on efficiency and a preference for certainty, with industry resources directed more toward screening and optimizing known targets, commercially attractive indications, and existing pipelines than toward exploring unknown disease mechanisms and novel intervention targets. This tendency stems partly from the high costs, substantial risks, long development cycles, and high failure rates inherent in drug development and is further reinforced by capital-market preferences, clinical translation windows, and commercialization pressures (DiMasi et al., 2016; Wouters et al., 2020). Existing research suggests that AI can substantially enhance data processing, molecular screening, and candidate optimization in drug discovery. Nevertheless, whether these capabilities can be translated into clinically effective and radically innovative outcomes still depends on an adequate understanding of disease mechanisms, appropriate target selection, and the quality of subsequent validation (Jayatunga et al., 2022; Paul et al., 2021). The central dilemma of radical innovation in AI-enabled drug discovery is therefore that, although AI has entered the drug development process, it may be used primarily to accelerate existing tasks and optimize established solutions rather than to redefine scientific problems, extend knowledge boundaries, and generate potentially radical R&D hypotheses (Jayatunga et al., 2022).

This dilemma suggests that realizing the radical innovation potential of AI in drug development depends not only on whether firms adopt or possess advanced AI technologies but, more importantly, on how R&D employees invoke, interpret, integrate, and further process AI-generated outputs in specific research activities. Employee creativity constitutes a microfoundation of organizational innovation, particularly in the form of radical creativity. Unlike general or incremental creativity, radical creativity emphasizes employees’ generation of highly novel ideas that deviate substantially from existing practices and possess the potential to produce breakthroughs (Amabile, 2020; Madjar et al., 2011). As AI has become increasingly integrated into knowledge work and innovation activities, its relationship with employee creativity has emerged as an important topic in management and information systems research. However, existing studies have not reached a consistent conclusion. On the one hand, AI can enhance creative performance by providing informational cues, generating alternative solutions, expanding the scope of search, and facilitating human–AI collaboration (Boussioux et al., 2024; Yin et al., 2024). On the other hand, AI may undermine employees’ independent judgment and creative engagement by inducing algorithmic dependence, cognitive offloading, idea homogenization, and task displacement (Doshi and Hauser, 2024; Raisch and Krakowski, 2021). Therefore, the effect of AI on radical creativity may depend less on the intensity of AI use itself than on the types of knowledge activities for which employees use it—whether they leverage AI to explore unknown problems and create novel knowledge combinations or merely use it to screen and optimize existing tasks and improve efficiency (Dell’Acqua et al., 2026; Krakowski, 2025).

Drawing on the exploration–exploitation framework, this study distinguishes between exploratory and exploitative AI use among employees. Exploratory AI use reflects distant knowledge search and refers to employees’ use of AI to understand disease mechanisms, identify unknown problems, discover novel targets, integrate knowledge across domains, and generate unconventional R&D hypotheses. Its core purpose is to extend employees’ cognitive boundaries and uncover latent knowledge associations through AI (March, 1991; Rosenkopf and Nerkar, 2001). In the context of AI-enabled drug discovery, exploratory AI use is more closely aligned with the upstream cognitive logic of identifying appropriate intervention targets for complex diseases. It can help employees move beyond the constraints of established targets and existing pipelines, thereby increasing the likelihood of radical creativity (Acar et al., 2024; Kaplan and Vakili, 2015). In contrast, exploitative AI use reflects local knowledge search and refers to employees’ use of AI for data processing, solution screening, property prediction, experimental optimization, and process improvement within existing R&D tasks. Its primary purpose is to enhance efficiency, reduce uncertainty, and accelerate progress toward predefined objectives (Balasubramanian et al., 2022; Benner and Tushman, 2003). Although exploitative AI use can improve short-term R&D efficiency, excessive reliance on existing data, established trajectories, and quantifiable outcomes may reinforce local search and path dependence, thereby reducing employees’ engagement with unknown disease mechanisms, unconventional knowledge combinations, and entirely new research directions (Levinthal and March, 1993; Lyell and Coiera, 2017). Distinguishing between exploratory and exploitative AI use therefore helps explain why AI may function either as a cognitive partner that facilitates radical creativity in pharmaceutical innovation or as an instrument for local optimization and efficiency-driven competition.^1^

Furthermore, how employees use AI is determined not solely by their individual choices but also by leaders’ attention to changes in the external environment and the behavioral direction they provide. Leaders are not merely recipients of external environmental information; they can also shape employees’ behavioral orientations through goal setting, resource allocation, task assignments, and performance requirements (Nadkarni and Barr, 2008; Ocasio, 1997). Accordingly, this study distinguishes between leader-perceived technological dynamism and leader-perceived market dynamism. Leader-perceived technological dynamism emphasizes rapid changes in AI algorithms, data resources, computing platforms, and R&D tools. Such changes expand the potential knowledge-search space and increase the need for learning about and experimenting with new technologies, thereby exhibiting a stronger functional fit with exploratory AI use (Haefner et al., 2021; Helfat and Peteraf, 2015). Leader-perceived market dynamism emphasizes rapid changes in capital preferences, competitive conditions, clinical needs, and commercialization windows. These changes increase the importance of timely responses, reliable delivery, and shorter R&D cycles and therefore exhibit a stronger functional fit with exploitative AI use (Calantone et al., 2003; Jaworski and Kohli, 1993). Importantly, neither technological nor market dynamism has an inherently positive or negative valence, as environmental changes can simultaneously present challenges and opportunities (Li et al., 2025; Malik et al., 2024). Rapid technological change can expand the knowledge-search space, but it may also increase uncertainty surrounding technological trajectories, intensify competitive pressures, and accelerate the obsolescence of existing knowledge and capabilities (Meier and Kock, 2024; Kruse et al., 2026). Similarly, rapid market change can increase response and delivery pressures while also creating new clinical needs and commercial opportunities (Jones et al., 2024; Malik et al., 2024). Thus, this study does not equate technological dynamism with opportunity or market dynamism with threat. Instead, it argues that technological dynamism more directly highlights the need for knowledge updating and open search, whereas market dynamism more directly highlights the need for timely responses and process optimization. Leader temporal myopia increases the emphasis placed on short-term, predictable, and quantifiable outcomes and may therefore weaken the relationship between leader-perceived technological dynamism and exploratory AI use while strengthening the relationship between leader-perceived market dynamism and exploitative AI use (Lefebvre, 2024; Agnihotri et al., 2024, 2025). On this basis, we develop a moderated mediation model linking leaders’ perceptions of environmental dynamism, employees’ modes of AI use, and employee radical creativity.

This study seeks to make three contributions. First, it moves beyond the intensity of AI use to focus on the functional purposes and knowledge-search orientations underlying such use. By distinguishing between exploratory and exploitative AI use, this study examines their differential relationships with radical creativity when employees use AI either to search for distant new knowledge or to refine existing knowledge. Importantly, this distinction concerns employees’ functional use of AI in specific R&D activities rather than the exploration–exploitation orientation of the R&D project itself. Second, this study conceptualizes leader-perceived technological dynamism and leader-perceived market dynamism as two external environmental conditions that highlight distinct innovation and response demands. It explains how these conditions may guide employees toward exploratory or exploitative AI use, thereby extending research on AI and creativity by directing greater attention to its external environmental antecedents (Mikalef et al., 2019; Newkirk and Lederer, 2006). Third, this study identifies leader temporal myopia as an important boundary condition. It reveals how leaders’ emphasis on short-term performance weakens the positive relationship between leader-perceived technological dynamism and exploratory AI use while strengthening the positive relationship between leader-perceived market dynamism and exploitative AI use. In doing so, this study extends research on modes of AI use beyond a sole focus on external environmental antecedents by demonstrating the joint role of environmental conditions and leaders’ temporal orientation.

## 2 Theoretical foundation and hypotheses

### 2.1 Theoretical foundation

The cognitive appraisal perspective suggests that decision makers’ attention to and interpretation of environmental conditions may influence their behavioral choices (Lazarus and Folkman, 1984; Dutton and Jackson, 1987). The perceived technological and market dynamism examined in this study are not discrete events occurring at a particular point in time. Rather, they capture leaders’ overall judgments of the rate and unpredictability of relevant environmental changes over a given period and therefore reflect time-varying environmental conditions or patterns of change (Wilden and Gudergan, 2015; Jung et al., 2024). However, environmental conditions do not possess fixed challenge or threat valences, as the same condition may be appraised differently depending on individuals’ resources and the specific context (Podsakoff et al., 2023; Kunzelmann et al., 2025). Perceived environmental dynamism is therefore conceptually distinct from challenge and threat appraisals, which constitute separate constructs and require direct measurement (Searle and Auton, 2015). Because this study does not directly measure challenge or threat appraisals, it does not test a specific cognitive appraisal mechanism or infer leaders’ appraisals from employees’ subsequent behaviors. Instead, we draw only on the general proposition of the cognitive appraisal perspective that decision makers’ interpretations of environmental conditions may be associated with their behavioral choices (Bullock et al., 2010).

Environmental dynamism reflects the rate and unpredictability of changes in external technological, market, and competitive conditions over a given period and may heighten the demands for organizational learning, knowledge updating, and behavioral adaptation (Wilden and Gudergan, 2015; Jung et al., 2024). A high level of leader-perceived technological dynamism indicates that, during the period considered by the leader, new algorithms, data resources, and R&D tools are changing rapidly and their developmental trajectories are difficult to predict. Recent advances in deep learning, molecular databases, virtual screening, and generative molecular design in AI-enabled drug discovery further illustrate this characteristic (Sadybekov and Katritch, 2023; Tropsha et al., 2024; Catacutan et al., 2024). On the one hand, these advances expand the chemical and knowledge spaces available for search, thereby increasing the importance of experimenting with new methods and identifying new R&D opportunities (Sadybekov and Katritch, 2023; Catacutan et al., 2024). On the other hand, rapid technological change may also increase uncertainty surrounding technological trajectories and application outcomes, intensify competition between existing and emerging technologies, and increase the risk that established products, technology platforms, and knowledge bases will become obsolete (Chandrasekaran et al., 2022; Jung et al., 2024).

Similarly, market dynamism may increase the need for organizations to identify and respond promptly to changing demands, execute tasks reliably, and optimize existing processes, while also revealing new customer needs and market opportunities (Homburg and Wielgos, 2022; Lendowski et al., 2023; Jung et al., 2024). Therefore, neither technological nor market dynamism has an inherently fixed positive or negative valence, nor can they be directly equated with opportunity and threat appraisals, respectively. The same environmental demands may be appraised differently depending on individuals’ resources and the specific context (Podsakoff et al., 2023; Kunzelmann et al., 2025). In the context of AI-enabled drug discovery, this study distinguishes between these two forms of environmental dynamism because technological dynamism more directly highlights the need for knowledge updating, technological learning, and open search, whereas market dynamism more directly highlights the need for demand responsiveness, reliable execution, and efficiency improvement (Wilden and Gudergan, 2015; Jansen et al., 2006; Lendowski et al., 2023). Accordingly, we propose that these two patterns of environmental change may exhibit relationships with exploratory and exploitative AI use that differ in emphasis. However, we do not suggest that either form of environmental dynamism can give rise exclusively to only one mode of AI use (Jansen et al., 2006; O’Reilly III and Tushman, 2013). Building on this distinction, we develop our hypotheses along two parallel pathways. First, we examine how leaders’ perceptions of technological dynamism are positively associated with employee radical creativity through employees’ exploratory use of AI, and how leader temporal myopia attenuates this positive indirect relationship. Second, we investigate how leaders’ perceptions of market dynamism are negatively associated with employee radical creativity through employees’ exploitative use of AI, and how leader temporal myopia amplifies this negative indirect relationship.

### 2.2 Leader perceptions of technological dynamism, exploratory AI use, and employee radical creativity

Leaders’ perceptions of technological dynamism capture their overall assessment of the speed, magnitude, and unpredictability of changes in AI algorithms, data resources, computing platforms, and R&D tools over a given period (Czakon et al., 2023a, 2023b; Keding, 2021). The rapid evolution of AI algorithms, data resources, and R&D tools has dual implications. On the one hand, these advances can integrate biomedical data with relevant background knowledge, thereby expanding the knowledge search space for elucidating disease mechanisms, generating R&D hypotheses, and identifying novel therapeutic targets (Cory-Wright et al., 2024; Zhang et al., 2025; Pun et al., 2026). On the other hand, they may also create risks associated with data quality, model generalizability, the selection of technological trajectories, and the accelerated obsolescence of existing knowledge (Messeri and Crockett, 2024; Bender et al., 2026).

Because the same environmental demand may elicit different or even coexisting cognitive appraisals (Körner et al., 2025), we do not *a priori* characterize technological dynamism as a challenge stressor, nor do we examine any specific cognitive appraisal mechanism. Instead, we develop our hypothesis based on the functional alignment between environmental action demands and modes of AI use. When technologies change rapidly and their future trajectories are difficult to predict, organizations face heightened demands for knowledge updating, experimentation, and the search for alternative solutions. Exploratory AI use is well suited to addressing these demands because it facilitates open-ended search, the mobilization of cross-domain knowledge, and the identification of previously unrecognized problems (Levinthal and Schliesmann, 2025; Boussioux et al., 2024). Accordingly, leaders’ perceptions of technological dynamism are likely to be positively associated with employees’ exploratory use of AI.

*Hypothesis 1*: Leaders’ perceptions of technological dynamism are positively related to employees’ exploratory use of AI.

Exploratory AI use refers to employees’ use of AI for open-ended knowledge search, cross-domain knowledge integration, the identification of previously unrecognized problems, and the generation of novel solutions. Its defining feature lies in leveraging AI to extend employees’ cognitive boundaries and uncover non-obvious connections among disparate bodies of knowledge (Jarrahi et al., 2023). Here, the term exploratory refers to the knowledge-search purpose underlying employees’ use of AI, rather than to the overall nature of the R&D projects in which they are engaged. AI use is considered exploratory in this study only when employees actively leverage AI to expand existing problem spaces, broaden the range of knowledge sources consulted, or identify alternative technological trajectories. In the context of AI-enabled drug discovery, exploratory AI use can help employees identify potential connections across biomedical literature, omics data, chemical structures, and clinical information, thereby supporting the elucidation of disease mechanisms, the discovery of novel therapeutic targets, and the generation of R&D hypotheses (Dara et al., 2022; Wong et al., 2023). Radical creativity refers to employees’ generation of ideas that are highly novel, depart substantially from existing practices, and possess the potential to produce breakthrough outcomes. Its development typically depends on broad knowledge search, problem reframing, and the recombination of heterogeneous knowledge (Liu et al., 2024). Accordingly, when employees use AI to explore new problems and recombine knowledge across disciplinary boundaries, they are more likely to move beyond established R&D trajectories and generate radical ideas (Wong et al., 2023).

More specifically, exploratory AI use may be associated with greater radical creativity through three mechanisms. First, AI can broaden the scope of employees’ information search, exposing them to a wider range of interdisciplinary and unconventional knowledge and thereby increasing the likelihood of novel knowledge combinations (Jarrahi et al., 2023). Second, by generating alternative solutions, simulating potential outcomes, and providing immediate feedback, AI can help employees redefine the problem space and identify new solution pathways (Epstein et al., 2023; Girotra et al., 2023). Third, exploratory AI use involves employees’ active evaluation, interpretation, and further refinement of AI-generated outputs. Such active engagement can mitigate the homogenization of ideas that may arise from the uncritical acceptance of algorithmic suggestions and enable employees to transform AI-generated content into more distinctive creative solutions (Doshi and Hauser, 2024). Taken together, we expect exploratory AI use to be positively related to employee radical creativity.

*Hypothesis 2*: Employees’ exploratory use of AI is positively related to their radical creativity.

Leader temporal myopia reflects a cognitive tendency to favor immediate, quantifiable, and predictable outcomes in intertemporal decision-making, which may lead leaders to allocate attention and innovation-related resources primarily according to short-term performance considerations (Ocasio, 1997; Wiersema et al., 2025). When leaders perceive rapid changes in AI algorithms, data resources, and R&D tools, technological dynamism heightens the organizational need to update knowledge, experiment with new tools, and search for alternative solutions (Wilden and Gudergan, 2015; Chen et al., 2018). Whether such environmental signals are translated into employees’ exploratory use of AI, however, depends partly on how leaders interpret the temporal costs and return structure of exploratory activities.

Exploratory AI use typically requires employees to devote substantial time to open-ended search, cross-domain knowledge integration, and repeated experimentation. These activities are characterized by a relatively high probability of failure, interim outcomes that are difficult to quantify, and benefits that may take considerable time to materialize (March, 1991; Levinthal and March, 1993; Agrawal et al., 2018; Ahmadi et al., 2025). Leaders with greater temporal myopia are therefore more likely to translate technological change into demands for the rapid adoption of proven tools, the timely completion of predefined R&D tasks, and the production of visible outcomes, rather than into sustained support for expanding problem spaces and experimenting with unconventional solutions. This tendency is consistent with the organizational learning argument that short-term feedback disproportionately reinforces exploitation while crowding out exploration (March, 1991; Levinthal and March, 1993; Liu et al., 2025).

Leaders can further communicate action priorities to employees through goal setting, task assignment, resource allocation, and performance evaluation, while their opening and closing leadership behaviors shape employees’ engagement in exploratory and exploitative activities (Ocasio, 1997; Rosing et al., 2011; Wilden et al., 2023). When these managerial signals are concentrated on near-term deliverables, employees may reduce the time and cognitive effort devoted to exploratory AI activities even when they face high levels of technological dynamism (Agrawal et al., 2018; Ahmadi et al., 2025; Joo et al., 2025). By contrast, leaders with lower temporal myopia are more tolerant of the uncertainty and delayed returns inherent in exploration and are more willing to preserve the time and resources required for new knowledge search, experimentation, and learning. Under such conditions, leaders’ perceptions of technological dynamism are more likely to be translated into employees’ exploratory use of AI (March, 1991; Agrawal et al., 2018). Accordingly, we propose that leader temporal myopia weakens the positive relationship between leaders’ perceptions of technological dynamism and employees’ exploratory use of AI.

*Hypothesis 3*: Leader temporal myopia negatively moderates the relationship between leaders’ perceptions of technological dynamism and employees’ exploratory use of AI, such that this positive relationship is weaker when leader temporal myopia is higher.

Furthermore, leader temporal myopia is expected to weaken the positive indirect relationship between leaders’ perceptions of technological dynamism and employee radical creativity through employees’ exploratory use of AI. Leaders’ perceptions of technological dynamism reflect their overall assessment of the pace and unpredictability of changes in AI algorithms, data resources, computing platforms, and R&D tools over a given period. In dynamic technological environments, the value of existing solutions may change rapidly, thereby requiring organizations to broaden their search scope, update their knowledge bases, and identify alternative technological options (Srikanth and Ungureanu, 2025). Exploratory AI use enables employees to respond to these demands by facilitating large-scale literature retrieval and knowledge synthesis, the search of complex solution spaces, and the generation of R&D hypotheses (Agrawal et al., 2024; Asai et al., 2026). Thus, leaders’ perceptions of technological dynamism and employees’ exploratory use of AI exhibit a high degree of functional alignment.

This functional alignment does not imply that technological dynamism is inherently a challenge stressor. Rapid technological change may also increase uncertainty surrounding technological choices, raise search costs, and heighten the risk that existing technologies will become obsolete (Srikanth and Ungureanu, 2025; Shi et al., 2025). At the same time, exploratory activities are generally characterized by substantial experimentation costs, a high probability of failure, lengthy feedback cycles, and difficulty in accurately assessing their value at an early stage. Innovations with the potential to generate substantial technological impact therefore require managerial patience and sustained investment (Shi et al., 2025).

Leader temporal myopia captures managers’ tendency to favor near-term outcomes, potentially at the expense of longer-term interests (Wiersema et al., 2025). When leader temporal myopia is high, leaders’ emphasis on immediate outputs and quantifiable performance may reduce their support for open-ended search, sustained experimentation, and long-term knowledge accumulation. Because leaders’ control over resources and the forms of support provided to employees can shape employees’ exploratory behavior (Evans and Schilke, 2024), such a short-term orientation is likely to inhibit the extent to which perceived technological dynamism stimulates exploratory AI use and, in turn, employee radical creativity. By contrast, leaders with lower temporal myopia are more likely to tolerate the uncertainty and delayed returns associated with exploration, thereby strengthening this positive indirect relationship (Shi et al., 2025; Wiersema et al., 2025). Accordingly, we propose:

*Hypothesis 4*: Leader temporal myopia moderates the positive indirect relationship between leaders’ perceptions of technological dynamism and employee radical creativity through employees’ exploratory use of AI, such that this positive indirect relationship is weaker when leader temporal myopia is higher.

### 2.3 Leader perceptions of market dynamism, exploitative AI use, and employee radical creativity

Leaders’ perceptions of market dynamism reflect their overall assessment of the pace and unpredictability of changes in clinical needs, investor preferences, competitors’ development pipelines, and commercialization windows over a given period (Kohli and Jaworski, 1990; Jaworski and Kohli, 1993; Calantone et al., 2003; Kang et al., 2024). When these market conditions change rapidly, the alignment between existing R&D plans and current market information may deteriorate within a relatively short period. Leaders must therefore update their judgments more frequently, compare alternative courses of action, and adjust resource allocations accordingly (Nadkarni and Barr, 2008; Teece, 2007; Wilden and Gudergan, 2015).

Market dynamism may increase the pressure associated with forecasting, responding to change, and reallocating resources, while also creating new demand and commercial opportunities (Calantone et al., 2003; Lendowski et al., 2023; Guhl et al., 2024). Regardless of whether leaders interpret particular market changes as a source of pressure or as an opportunity, promptly identifying such changes, mobilizing resources, and improving the responsiveness of existing R&D processes constitute important action requirements in dynamic market environments (Kohli and Jaworski, 1990; Teece, 2007). Exploitative AI use is functionally aligned with these requirements because exploitation emphasizes the refinement and improvement of existing knowledge and practices, as well as efficiency enhancement, within established task objectives and evaluation criteria (Jansen et al., 2006; Lendowski et al., 2023).

More specifically, exploitative AI use operates within the boundaries of existing task objectives, data resources, and evaluation criteria. It can help employees rapidly consolidate R&D- and project-related information, screen candidate solutions, predict compound properties, optimize experimental parameters, and prioritize tasks. In doing so, it reduces the time required for information processing and alternative comparison, lowers the costs of repetitive experimentation, and enhances the controllability of the R&D process (Vamathevan et al., 2019; Paul et al., 2021; Sadybekov and Katritch, 2023; Edfeldt et al., 2024; Zhang et al., 2025).

In the context of AI-enabled drug discovery, the more strongly leaders perceive market dynamism, the more likely they are to emphasize shortening R&D cycles, improving resource-use efficiency, and advancing candidate projects with near-term translational potential. Through goal setting, attention allocation, and resource deployment, leaders communicate these action priorities to employees (Ocasio, 1997; Nadkarni and Barr, 2008; Wilden et al., 2023). Under such conditions, employees are more likely to use AI for prediction, screening, and optimization within established R&D trajectories to accelerate decision-making and task execution (Kolev et al., 2025; Zhang et al., 2025). Accordingly, stronger leader perceptions of market dynamism are more likely to translate into greater employee exploitative AI use.

*Hypothesis 5*: Leaders’ perceptions of market dynamism are positively related to employees’ exploitative use of AI.

Exploitative AI use refers to employees’ use of AI for data processing, alternative screening, property prediction, process optimization, and experimental iteration within established R&D tasks. Its defining purpose is to enhance efficiency, reduce uncertainty, and accelerate progress toward predefined objectives (Brynjolfsson et al., 2025; Noy and Zhang, 2023). Here, the term exploitative describes the functional purpose of using AI to refine existing knowledge, optimize current solutions, and improve predefined tasks; it does not imply that all AI use in mature-stage R&D projects should automatically be classified as exploitative. Existing empirical evidence indicates that generative AI can substantially improve knowledge workers’ task-completion speed and productivity, but that these benefits are particularly pronounced for tasks that are structured, decomposable, and clearly defined (Dell’Acqua et al., 2026). In the context of AI-enabled drug discovery, exploitative AI use can accelerate candidate compound screening, experimental parameter optimization, and pipeline advancement. Nevertheless, its primary function remains the incremental improvement of existing solutions within predefined R&D objectives, rather than the redefinition of problems or the exploration of unconventional directions (Jayatunga et al., 2022; Vora et al., 2023). Radical creativity, by contrast, involves the generation of ideas that are highly novel, depart substantially from existing practices, and have the potential to produce breakthrough value. Such creativity relies more heavily on distant knowledge search, problem reframing, and the recombination of heterogeneous knowledge (Acar et al., 2024). Thus, although exploitative AI use may enhance short-term efficiency, it may impede employees’ generation of radical ideas.

More specifically, exploitative AI use may be associated with lower radical creativity by reinforcing path dependence, constraining deep reflection, and inducing overreliance on automation. First, because exploitative AI use is typically embedded within established task objectives and data frameworks, it may reinforce employees’ reliance on mature targets, predefined procedures, and existing evaluation criteria, thereby reducing their exploration of unknown mechanisms and unconventional R&D hypotheses (Balasubramanian et al., 2022). Second, the emphasis of exploitative AI use on rapid prediction, screening, and optimization may direct employees’ attention toward the immediate usability of AI-generated outputs at the expense of deeper reflection on the underlying nature of the problem and possible alternative pathways (Dell’Acqua et al., 2026). Finally, persistent reliance on AI to perform predefined tasks may foster automation bias, causing employees to accept AI recommendations excessively while reducing their proactive information search and independent judgment (Abdelwanis et al., 2024; Lyell and Coiera, 2017). Consequently, as employees engage more extensively in exploitative AI use, they may increasingly regard AI as an efficiency-enhancing tool rather than as a cognitive partner, which may ultimately be associated with lower radical creativity.

*Hypothesis 6*: Employees’ exploitative use of AI is negatively related to their radical creativity.

Leaders’ perceptions of market dynamism do not necessarily lead employees to adopt a particular mode of AI use. Market dynamism primarily changes the informational and response demands confronting an organization, whereas whether these demands ultimately give rise to exploration or exploitation depends on how managers allocate limited attention and resources between the two types of activity (Jansen et al., 2006; Walrave et al., 2017; Lendowski et al., 2023). In the face of shifting customer needs, intensifying competition, and narrowing commercialization windows, leaders may preserve future options through long-term knowledge search and capability development, or they may pursue more immediate outcomes by optimizing existing solutions and improving execution efficiency (March, 1991; O’Reilly III and Tushman, 2013; Walrave et al., 2017). Leader temporal myopia alters the relative priority assigned to these two response strategies. Leaders with greater temporal myopia place greater emphasis on near-term performance, short-term deliverables, and outcome predictability. They are therefore more likely to translate external pressures into exploitation-oriented demands, such as efficient execution, the attainment of predefined objectives, and adherence to established procedures (Levinthal and March, 1993; Aksoy, 2025; Liu et al., 2025; Wiersema et al., 2025). Compared with exploratory activities, which involve greater outcome uncertainty and longer payoff horizons, exploitative activities focus on refining existing knowledge, capabilities, and task processes and can therefore generate more immediate and relatively predictable performance returns. Such activities are consequently more consistent with the preferences of temporally myopic decision-makers (March, 1991; Vlašić et al., 2024).

More specifically, exploitative AI use can generate relatively observable outputs within established R&D objectives, including candidate compound prioritization, property prediction, virtual screening, experimental parameter optimization, and R&D process improvement. Its task boundaries, evaluation criteria, and expected payoff horizons are also comparatively well defined (Vamathevan et al., 2019; Paul et al., 2021; Sadybekov and Katritch, 2023; Zhang et al., 2025). Thus, when leaders with high temporal myopia perceive substantial market dynamism, they are more likely to translate external changes into direct pressure on employees to use AI to optimize existing solutions by shortening task cycles, tightening interim performance targets, prioritizing resource allocation, and raising delivery expectations. Prior research suggests that challenging performance goals can induce decision-makers to curtail continued exploration and increase their reliance on exploitative choices (Raveendran et al., 2023). Managers can also translate their interpretations of environmental issues into concrete employee actions through attention direction, goal communication, and resource allocation (Ocasio, 1997; Walrave et al., 2017). Leader temporal myopia therefore strengthens the functional alignment between perceived market dynamism and exploitative AI use.

By contrast, leaders with lower temporal myopia retain greater latitude to balance immediate responsiveness against long-term capability development. They may respond to market changes by exploring emerging needs, developing new technological options, and conducting forward-looking experiments. Consequently, their perceptions of market dynamism are less likely to be translated predominantly into exploitative AI use (O’Reilly III and Tushman, 2013; Walrave et al., 2017). Accordingly, we propose that the positive relationship between leaders’ perceptions of market dynamism and employees’ exploitative use of AI becomes stronger as leader temporal myopia increases.

*Hypothesis 7*: Leader temporal myopia positively moderates the relationship between leaders’ perceptions of market dynamism and employees’ exploitative use of AI, such that this positive relationship is stronger when leader temporal myopia is higher.

Furthermore, we propose that leader temporal myopia strengthens the negative indirect relationship between leaders’ perceptions of market dynamism and employee radical creativity through employees’ exploitative use of AI. In high-velocity environments, timely decision-making, rapid implementation, and responsiveness to competitive change become increasingly important for organizational performance. Accordingly, stronger perceptions of market dynamism are likely to accentuate action demands related to timely response, process optimization, and shorter R&D cycles (Kolev et al., 2025). AI-enabled computational methods can improve the efficiency of compound property prediction, virtual screening, candidate prioritization, and lead optimization, creating a high degree of functional alignment between exploitative AI use and these action demands (Lam and Katritch, 2025).

However, although excessive reliance on existing knowledge and local search may generate relatively rapid short-term returns, it can also foster knowledge lock-in and constrain broader technological diffusion. Compared with cross-domain search, the intensive exploitation of existing technologies is also more likely to produce incremental rather than radical innovation (Harrison et al., 2024; Cao and Evans, 2026). Managerial myopia may further narrow the breadth of knowledge underlying patents, reduce the technological content of innovation, and orient R&D activities under external pressure toward quantity rather than quality (Yu et al., 2024). Thus, when leader temporal myopia is high, leaders are more likely to concentrate attention and resources on exploitative AI use characterized by lower risk, shorter payoff horizons, and more readily quantifiable outcomes. This tendency, in turn, strengthens the negative indirect relationship between leaders’ perceptions of market dynamism and employee radical creativity through employees’ exploitative use of AI. Accordingly, we propose:

*Hypothesis 8*: Leader temporal myopia moderates the negative indirect relationship between leaders’ perceptions of market dynamism and employee radical creativity through employees’ exploitative use of AI, such that this negative indirect relationship is stronger—that is, more negative—when leader temporal myopia is higher (see Figure 1).

**Figure 1:**
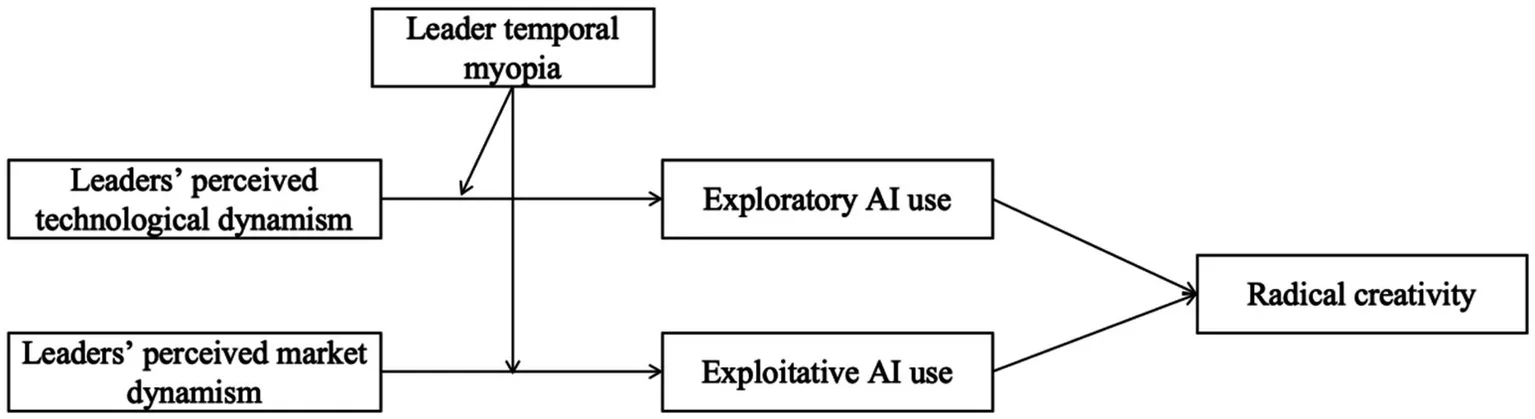
Theoretical model. H4 and H8 denote the hypothesized moderating effects of leader temporal myopia on the indirect effects operating through exploratory and exploitative AI use, respectively.

## 3 Research design

### 3.1 Sample and data collection

To ensure the reliability and validity of the measurement instrument, this study first drew on established literature to identify measurement items for all focal variables and then adapted them to the employee-level context of AI-enabled drug R&D. The exploratory and exploitative AI use scales were adapted according to three principles. First, references to organizational innovation activities in the original scale developed by Jansen et al. (2006) were reformulated as employee-level AI use behaviors. Second, the core distinction in the original scale between the search for distant new knowledge and the refinement of existing knowledge was retained. Third, behavioral anchors specific to AI-enabled drug R&D were incorporated. Specifically, the two knowledge-search orientations were operationalized as employees’ use of AI to identify new problems, integrate cross-domain knowledge, and generate unconventional hypotheses, and their use of AI to screen alternative solutions, predict properties, optimize processes, and enhance efficiency, respectively. These contextualized descriptions were intended to clarify the functional purpose for which employees used AI, rather than to classify a project as exploratory or exploitative solely on the basis of the name or type of its R&D task. On this basis, an English-language questionnaire was developed. Graduate students holding professional English translation credentials were then invited to translate the English items into Chinese and verify the semantic equivalence of the English and Chinese versions. Subsequently, the questionnaire was pretested with 30 biotechnology firms in Guangdong Province. Based on the pretest results, minor revisions were made to the wording of several items, resulting in the final Chinese-language questionnaire.

This study used purposive sampling to identify eligible participants. The initial list of firms was compiled primarily from the official websites of the National Medical Products Administration and other relevant government agencies and covered provinces in eastern China. Drawing on publicly available corporate information, official company websites, business descriptions, and preliminary communications, the research team first identified firms that appeared to be substantively engaged in AI-enabled drug R&D. Corporate contacts were then asked to confirm the firms’ business activities and relevant project involvement. WeChat, QQ, and email were used solely as channels for contacting firms and distributing questionnaires; they did not constitute independent sampling frames. Because the sample was drawn from firms that were both willing to participate and able to provide matched leader–employee data, it represents a nonprobability purposive sample and may therefore be subject to self-selection bias.

To ensure that participating firms and respondents fit the research context, the following inclusion criteria were applied. First, firms had to be substantively engaged in drug discovery, drug development, biotechnology R&D, or related R&D services, rather than being involved solely in pharmaceutical sales or general administrative activities. Second, firms had to be using AI in at least one stage of the drug R&D process, including target identification, disease-mechanism analysis, molecular generation, virtual screening, property prediction, candidate compound optimization, or preclinical development. Third, participating employees had to be directly involved in these AI-enabled R&D activities and have at least 3 months of relevant AI use experience. Fourth, participating leaders had to be directly responsible for the focal employee’s task assignment, project management, or performance evaluation and had to have worked with that employee for at least 12 months. Firms and respondents who used general-purpose generative AI only for document processing, information retrieval, or routine office work, without applying AI to drug R&D tasks, were excluded from the formal sample. Eligibility was verified through screening questions, confirmation by corporate contacts, publicly available corporate information, or descriptions of relevant projects.

For each eligible firm, the research team selected one team that was actively engaged in AI-enabled drug R&D and invited one leader directly responsible for the team or project and one employee who used AI in conducting R&D activities to form a matched dyad. Only one leader–employee dyad was retained from each firm. Leaders and employees completed separate questionnaires, and their responses were matched across sources and survey waves using anonymous identification codes. Importantly, the use of one dyad per firm was intended to produce leader–employee observations that were independent across firms; it does not imply that the selected dyad was representative of the firm or team as a whole.

Before questionnaire administration, potential respondents were informed that participation was restricted to team leaders and R&D personnel working in AI-enabled drug R&D teams. To reduce potential method bias arising from measuring variables at the same time and from the same source, while ensuring that the measurement sequence was consistent with the ordering specified in the theoretical model, this study employed a three-wave, multisource, matched leader–employee survey design, with a two-week interval between adjacent waves. The two-week interval was selected for two main reasons. First, this study focuses on relatively proximal cognitive and behavioral manifestations, such as employees’ use of AI for knowledge search and solution generation and their production of original R&D ideas, rather than long-term innovation outcomes such as drug approval, clinical translation, or commercialization. These focal behaviors may be observable over relatively short periods during routine R&D activities. Second, a relatively short interval helps maintain consistency in the project context across survey waves and reduces attrition in a multiwave survey. Nevertheless, the two-week interval was used primarily to separate the measurement occasions. It should not be interpreted as the optimal time window for identifying causal effects, nor can it capture the full process of long-term knowledge accumulation and repeated experimentation in AI-enabled drug R&D. Moreover, because employees’ AI use modes and radical creativity were not measured repeatedly across multiple time points, the research design provides temporally separated correlational evidence whose measurement order is consistent with the theoretical model, but it cannot, by itself, establish strict causal relationships.

In Wave 1, team leaders reported their perceptions of technological dynamism, perceptions of market dynamism, temporal myopia, and relevant team- and firm-level characteristics. Employees reported their demographic information, AI literacy, and the current development stage of their focal projects. A total of 600 leader–employee dyads were initially invited to participate. The research team received 474 leader questionnaires and 523 employee questionnaires, of which 437 dyads had completed both questionnaires and could be successfully matched. Two weeks later, the employees in these matched dyads were invited to complete measures of exploratory and exploitative AI use, yielding 409 valid responses that could be matched with the Wave 1 data. After another two-week interval, the corresponding leaders were asked to evaluate the focal employees’ radical creativity, resulting in 393 dyads that could be matched across all three waves.

Following the three-wave matching procedure, questionnaires were further excluded if they had been completed in less than 5 min, contained substantial missing data, exhibited straight-lining across a large number of items, or had leader–employee identification codes that could not be matched accurately. The final sample consisted of 368 valid leader–employee dyads from 368 firms. Relative to the 437 matched dyads obtained in Wave 1, the final retention rate was 84.21%.

The unit of analysis in this study is the leader–employee dyad. Leaders reported their perceptions of environmental dynamism and temporal myopia, employees reported their modes of AI use, and the corresponding leaders evaluated employees’ radical creativity. Because only one dyad was included from each firm, the focal variables were not aggregated to the team or firm level, and the study cannot identify within-team or within-firm variation. Accordingly, the empirical findings pertain only to relationships among variables at the dyadic level and should not be interpreted as evidence that team- or firm-level mechanisms have been tested.

### 3.2 Measures

The measurement items for all focal constructs are presented in see Table 1. All measurement items were rated on a five-point Likert scale ranging from 1 (“strongly disagree”) to 5 (“strongly agree”).

1. Independent variables: Leaders’ perceptions of technological dynamism (TD) and market dynamism (MD)

**Table 1:** Measurement items.

Type	Construct	Item
Independent	Technological dynamism	I believe that technologies related to AI-enabled drug discovery are developing and changing rapidly.
		I believe that the direction of AI-related technological development in drug discovery over the next 2 to 3 years is difficult to predict accurately.
		I believe that major breakthroughs in AI technologies are generating many new R&D ideas, approaches to target discovery, and drug-design solutions in AI-enabled drug discovery.
	Market dynamism	I believe that the preferences of customers, investors, or partners in AI-enabled drug discovery change substantially over time.
		I believe that customers, investors, or partners in AI-enabled drug discovery are continually seeking new R&D directions, product opportunities, or partnership opportunities.
		I believe that new R&D demands are emerging from new customers, investors, or partners in AI-enabled drug discovery.
		I believe that new customers, investors, or partners generally have R&D needs that differ from those of existing customers or partners.
Moderator	Temporal myopia	To achieve short-term R&D performance goals, I reduce investment in employees’ long-term capability development.
		To achieve short-term R&D performance goals, I reduce investment in long-term AI-enabled drug R&D projects.
		To achieve short-term R&D performance goals, I reduce investment in R&D activities that offer long-term returns but uncertain short-term outcomes.
		To achieve short-term R&D performance goals, I reduce investment in identifying future market needs, partnership opportunities, and frontier R&D directions.
Mediator	Exploratory AI use	I use AI to search for new problems or opportunities beyond my team’s existing R&D paths.
		I use AI to connect knowledge from different disciplines, disease areas, or technological fields to develop new R&D ideas.
		I use AI to explore new targets, mechanisms, or indications that are not directly related to the current project but may lead to breakthroughs.
		I use AI to generate unconventional R&D hypotheses that depart from existing approaches.
		I use AI to identify new technological trajectories, research directions, or application scenarios beyond the current project.
		I use AI to discover potential R&D opportunities that are difficult to identify through conventional experience or existing data-analysis methods.
		I use AI to introduce new data, knowledge, or tools from external fields into my current R&D work.
	Exploitative AI use	I use AI to improve the execution of existing tasks in the current R&D project.
		I use AI to screen and optimize existing targets, molecules, or candidate compounds.
		I use AI to improve the efficiency of data analysis, property prediction, or experimental iteration in the current R&D process.
		I use AI to optimize existing technological trajectories or R&D solutions in the current project.
		I use AI to improve the efficiency, accuracy, or reliability of existing R&D tasks.
		I use AI to extend or deepen the application of existing R&D outcomes in the current project.
		I use AI to reduce the cost and time of trial and error in the current R&D process.
Dependent	Radical creativity	This employee is an important source of highly original R&D ideas.
		This employee demonstrates a high degree of originality in AI-enabled drug R&D.
		This employee proposes novel methods or solutions that differ substantially from established R&D paths.

All exploratory and exploitative AI use items presuppose that employees actually use AI. The R&D activities described in the items identify the functional purpose and knowledge-search orientation of AI use; they do not directly describe the overall attributes of the employee’s R&D project.

Leaders’ perceptions of technological dynamism and market dynamism were measured using contextually adapted versions of the scales developed by Jaworski et al. (1993). Leaders’ perceptions of technological dynamism were measured with three items, with a Cronbach’s α of 0.872. Leaders’ perceptions of market dynamism were measured with four items, with a Cronbach’s α of 0.892.

1. Mediating variables: Exploratory AI use (EA) and exploitative AI use (UA)

Exploratory and exploitative AI use were measured using scales adapted from Jansen et al. (2006) to the employee-level context of AI-enabled drug R&D. We conceptualized both constructs as purpose-oriented AI use behaviors. Exploratory AI use captures whether employees use AI to extend the boundaries of their existing knowledge search, whereas exploitative AI use captures whether employees use AI to refine existing knowledge, optimize current solutions, and improve predefined tasks. Each item began with the behavioral qualifier “I use AI to …,” thereby requiring employees to have actually used AI to perform the specified activity for the behavior to qualify as one of the AI use modes examined in this study. Exploratory AI use was measured with seven items and exhibited a Cronbach’s α of 0.882, whereas exploitative AI use was measured with seven items and exhibited a Cronbach’s α of 0.912.

Conceptually, exploratory and exploitative AI use are distinct from both the exploration–exploitation orientation of the R&D task itself and the intensity of AI use. R&D task orientation describes the overall characteristics of a project or work task, regardless of whether AI is used. AI use intensity refers to the frequency, duration, or amount of employees’ AI use. In contrast, the AI use modes examined in this study capture the functional purposes and knowledge-search processes for which employees employ AI in specific R&D activities. Different modes of AI use may coexist within the same R&D project, and project characteristics may influence employees’ choice of AI use mode. Such associations, however, do not imply that project characteristics and AI use modes are conceptually equivalent.

1. Dependent variable: Employee radical creativity (BC)

Employee radical creativity was measured using a three-item scale adapted from Madjar et al. (2011), with a Cronbach’s α of 0.873. It is important to note that radical creativity in this study refers to employees’ creative performance in proposing highly original R&D ideas, methods, and solutions, rather than long-term innovation outcomes such as successful drug discovery, clinical translation, or commercialization. Accordingly, the Wave 3 assessment captures leaders’ evaluations of employees’ creative performance rather than evaluations of the ultimate outcomes of the entire drug development process.

1. Moderating variable: Leader temporal myopia (ST)

Leader temporal myopia was measured using a four-item scale adapted from Czakon et al. (2023a,b), with a Cronbach’s α of 0.902.

1. Control variables

We controlled for several team- and organizational-level characteristics that may influence employees’ AI use modes and radical creativity. First, AI literacy may affect the ability to embed AI in R&D processes and engage in exploratory or exploitative AI use. We therefore measured AI literacy using the 12-item scale developed by Wang et al. (2023), which exhibited a Cronbach’s α of 0.950.

Second, team size and team age may influence teams’ information-processing capacity, organizational inertia, and propensity for innovation. Third, R&D investment intensity may determine whether a team has sufficient resources to support highly uncertain exploratory AI activities.

Finally, the development stage of the focal project may influence the types of tasks for which AI is used. Early-stage R&D is more likely to involve disease-mechanism analysis and target identification, whereas later-stage R&D is more likely to involve molecular optimization and pipeline advancement. We therefore included these variables as controls in the empirical models. Controlling for the project’s development stage helps account, to some extent, for task differences between early-stage activities, such as mechanism exploration and target identification, and later-stage activities, such as molecular optimization and pipeline advancement. However, the development stage cannot fully capture a project’s exploratory orientation, task novelty, or technological uncertainty. Thus, we cannot completely rule out the possibility that project characteristics simultaneously influence employees’ AI use modes and radical creativity.

## 4 Empirical results

### 4.1 Common method bias

First, Harman’s single-factor test was conducted to assess potential common method bias. The first unrotated principal component accounted for 32.358% of the total variance, which was below the conventional threshold of 40%, providing preliminary evidence that common method bias was unlikely to be a serious concern. Building on this analysis, we further applied the unmeasured latent method factor approach using the covariance-based structural equation modeling (CB-SEM) function in SmartPLS 4.0. Specifically, a model incorporating a common method factor was compared with the baseline six-factor model.

The baseline six-factor model demonstrated a good fit to the data (χ^2^ = 577.724, χ^2^/df = 1.725, CFI = 0.960, TLI = 0.955, and RMSEA = 0.044). Although adding the common method factor slightly improved the model fit (χ^2^ = 495.744, χ^2^/df = 1.615, CFI = 0.969, TLI = 0.962, and RMSEA = 0.041), the magnitude of improvement was limited. Given that the baseline six-factor model already exhibited a good fit and that the inclusion of the method factor did not result in a substantive improvement in model fit, common method bias was unlikely to have materially affected the study’s findings.

### 4.2 Confirmatory factor analysis

This study conducted covariance-based confirmatory factor analysis (CB-CFA) using the CB-SEM function in SmartPLS 4.0 to examine the factorial structure of the focal constructs and to compare the hypothesized six-factor model with several alternative measurement models. The results are presented in Table 2. It should be noted that CB-CFA was used in this section to assess the overall factorial structure of the measurement constructs, whereas PLS-SEM was subsequently employed to evaluate the measurement model and the hypothesized structural relationships. Thus, the two analytical approaches served distinct purposes in this study.

**Table 2:** Confirmatory factor analysis.

Model	χ^2^	df	χ^2^/df	CFI	TLI	RMSEA
Six-factor model (including a common method factor)	495.744	307	1.615	0.969	0.962	0.041
Six-factor model: TD; MD; ST; EA; UA; BC	577.724	335	1.725	0.960	0.955	0.044
Five-factor model: TD + MD; ST; EA; UA; BC	1096.129	340	3.224	0.877	0.863	0.078
Four-factor model: TD + MD; ST; EA + UA; BC	2061.858	344	5.994	0.720	0.693	0.117
Three-factor model: TD + MD + ST; EA + UA; BC	2950.345	347	8.502	0.576	0.538	0.143
Two-factor model: TD + MD + ST; EA + UA + BC	3398.351	349	9.737	0.503	0.462	0.154
One-factor model: TD + MD + ST + EA + UA + BC	3780.592	350	10.802	0.441	0.396	0.163

“+” indicates that two factors were combined into one. TD, leader-perceived technological dynamism; MD, leader-perceived market dynamism; ST, leader temporal myopia; EA, exploratory AI use; UA, exploitative AI use; BC, employee radical creativity.

The results indicated that the six-factor model provided a better overall fit than the competing models and satisfied the commonly recommended fit criteria: χ^2^/df was 1.725, below the threshold of 3; the CFI and TLI values were 0.960 and 0.955, respectively, both exceeding 0.90; and the RMSEA was 0.044, below 0.08. These results provide evidence that the six focal constructs are empirically distinguishable and exhibit satisfactory discriminant validity.

### 4.3 Measurement model

Following the preliminary assessment of the overall factor structure using CB-CFA, we further employed the PLS-SEM algorithm in SmartPLS 4.0 to evaluate the reliability and validity of the reflective measurement model. The results are presented in Tables 3, 4. First, the Cronbach’s α values for all constructs exceeded 0.80, and all composite reliability (CR) values were above 0.70, indicating a high level of internal consistency reliability. Second, all indicator loadings (λ) exceeded 0.60 and were statistically significant at *p* < 0.001. In addition, the average variance extracted (AVE) for each construct was above the recommended threshold of 0.50, providing evidence of satisfactory convergent validity.

**Table 3:** Reliability and convergent validity.

Construct	Item	λ	Cronbach’s α	CR	AVE
Leader-perceived technological dynamism	TD-1	0.902***	0.872	0.921	0.796
	TD-2	0.897***			
	TD-3	0.878***			
Leader-perceived market dynamism	MD-1	0.874***	0.892	0.925	0.756
	MD-2	0.849***			
	MD-3	0.843***			
	MD-4	0.910***			
Leader temporal myopia	ST-1	0.867***	0.902	0.932	0.773
	ST-2	0.887***			
	ST-3	0.847***			
	ST-4	0.915***			
Exploratory AI use	EA-1	0.794***	0.882	0.908	0.587
	EA-2	0.753***			
	EA-3	0.700***			
	EA-4	0.782***			
	EA-5	0.794***			
	EA-6	0.717***			
	EA-7	0.813***			
Exploitative AI use	UA-1	0.810***	0.912	0.930	0.654
	UA-2	0.810***			
	UA-3	0.777***			
	UA-4	0.833***			
	UA-5	0.794***			
	UA-6	0.829***			
	UA-7	0.805***			
Employee radical creativity	BC-1	0.891***	0.873	0.922	0.798
	BC-2	0.908***			
	BC-3	0.881***			

λ = standardized loading; ***p < 0.001; Cronbach’s α = Cronbach’s alpha coefficient; CR, composite reliability; AVE, average variance extracted.

**Table 4:** Square roots of AVE and latent-variable correlations.

Construct	1	2	3	4	5	6
Leader-perceived technological dynamism	**0.892**	*0.430*	*0.350*	*0.549*	*0.398*	*0.428*
Leader-perceived market dynamism	−0.380	**0.869**	*0.208*	*0.310*	*0.501*	*0.401*
Leader temporal myopia	−0.310	0.186	**0.879**	*0.464*	*0.180*	*0.323*
Exploratory AI use	0.482	−0.274	−0.414	**0.766**	*0.375*	*0.479*
Exploitative AI use	−0.355	0.451	0.164	−0.337	**0.809**	*0.392*
Employee radical creativity	0.373	−0.354	−0.286	0.421	−0.349	**0.893**

*N* = 368. Square roots of AVE are shown in bold on the diagonal; correlations are shown below the diagonal; and HTMT ratios are shown in italics above the diagonal.

Discriminant validity was subsequently assessed using the Fornell–Larcker criterion and the heterotrait–monotrait ratio of correlations (HTMT). The results showed that the square root of the AVE for each construct exceeded its correlations with the other constructs and that all HTMT values were below the recommended threshold of 0.85. These findings indicate satisfactory discriminant validity. More specifically, the HTMT value between exploratory AI use and exploitative AI use was 0.375, substantially below the 0.85 threshold, suggesting that respondents were able to distinguish empirically between the two modes of AI use.

It should be noted, however, that statistical discriminant validity primarily demonstrates that the two sets of measurement items do not constitute a single empirical construct. The distinction between AI use modes and the general exploration–exploitation orientation of R&D tasks must also be established by considering their different behavioral referents and objects of analysis. Accordingly, we do not treat statistical evidence of discriminant validity as a substitute for the preceding conceptual arguments concerning construct boundaries. Finally, variance inflation factor (VIF) values ranged from 1.166 to 1.935, and all tolerance values exceeded 0.100, indicating that multicollinearity was not a serious concern in the model.

### 4.4 Structural model

We used the bootstrapping procedure in SmartPLS 4.0 to estimate the structural model and test the proposed hypotheses. The results are reported in Table 5 and illustrated in Figure 2. For the exploratory AI use pathway, leaders’ perceptions of technological dynamism were positively related to employees’ exploratory use of AI (β = 0.456, *p* < 0.001), supporting Hypothesis 1. Employees’ exploratory use of AI was, in turn, positively related to employee radical creativity (β = 0.342, *p* < 0.001), supporting Hypothesis 2. In addition, leader temporal myopia significantly weakened the positive relationship between leaders’ perceptions of technological dynamism and employees’ exploratory use of AI (β = −0.118, *p* = 0.014), providing support for Hypothesis 3.

**Table 5:** Bootstrapping results.

Path	β	SE	t	p	95% confidence interval	f^2^
H1: TD → EA	0.456	0.043	10.699	<0.001	[0.371, 0.538]	0.224
H2: EA → BC	0.342	0.054	6.342	<0.001	[0.230, 0.441]	0.134
TD → BC	0.132	0.063	2.111	<0.05	[0.007, 0.252]	0.017
TD → EA → BC	0.156	0.029	5.339	<0.001	[0.099, 0.213]	—
ST → EA	−0.286	0.053	5.368	<0.001	[−0.390, −0.182]	0.109
H3: TD × ST → EA	−0.118	0.048	2.448	<0.05	[−0.212, −0.024]	0.022
H4: TD × ST → EA → BC	−0.040	0.017	2.302	<0.05	[−0.077, −0.008]	—
H5: MD → UA	0.399	0.057	6.952	<0.001	[0.283, 0.510]	0.191
H6: UA → BC	−0.239	0.050	4.792	<0.001	[−0.341, −0.142]	0.066
MD → BC	−0.170	0.052	3.245	<0.01	[−0.274, −0.065]	0.029
MD → UA → BC	−0.095	0.023	4.119	<0.001	[−0.146, −0.054]	—
ST → UA	0.082	0.050	1.635	>0.1	[−0.015, 0.181]	0.008
H7: MD × ST → UA	0.154	0.056	2.747	<0.01	[0.042, 0.259]	0.036
H8: MD × ST → UA → BC	−0.037	0.017	2.129	<0.05	[−0.075, −0.008]	—

β = path coefficient; SE = bootstrap standard error; TD = leader-perceived technological dynamism; MD = leader-perceived market dynamism; ST = leader temporal myopia; EA = exploratory AI use; UA = exploitative AI use; BC = employee radical creativity. f^2^ = effect size; ‘—’ indicates that this statistic is not applicable to an indirect effect.

**Figure 2:**
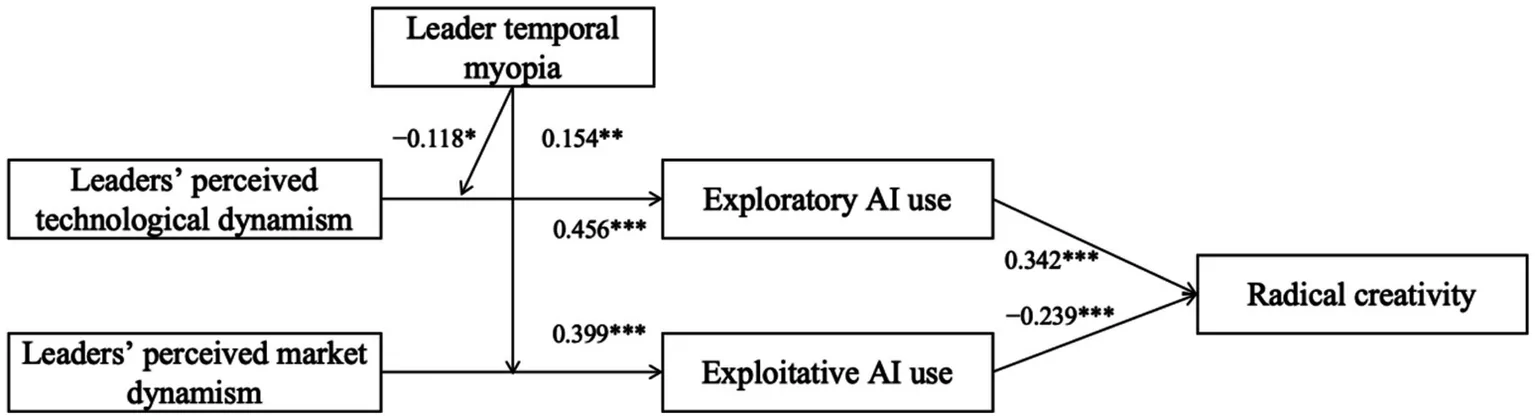
Path coefficients. *p < 0.05, **p < 0.01, ***p < 0.001. The moderated mediation effect for H4 was β = −0.040 (*p* = 0.021), whereas that for H8 was β = −0.037 (*p* = 0.033).

For the exploitative AI use pathway, leaders’ perceptions of market dynamism were positively related to employees’ exploitative use of AI (β = 0.399, *p* < 0.001), supporting Hypothesis 5. Employees’ exploitative use of AI was negatively related to employee radical creativity (β = −0.239, *p* < 0.001), supporting Hypothesis 6. Furthermore, leader temporal myopia significantly strengthened the positive relationship between leaders’ perceptions of market dynamism and employees’ exploitative use of AI (β = 0.154, *p* = 0.006), supporting Hypothesis 7. To further illustrate these moderating effects, we plotted the simple slopes, as shown in Figures 3, 4.

**Figure 3:**
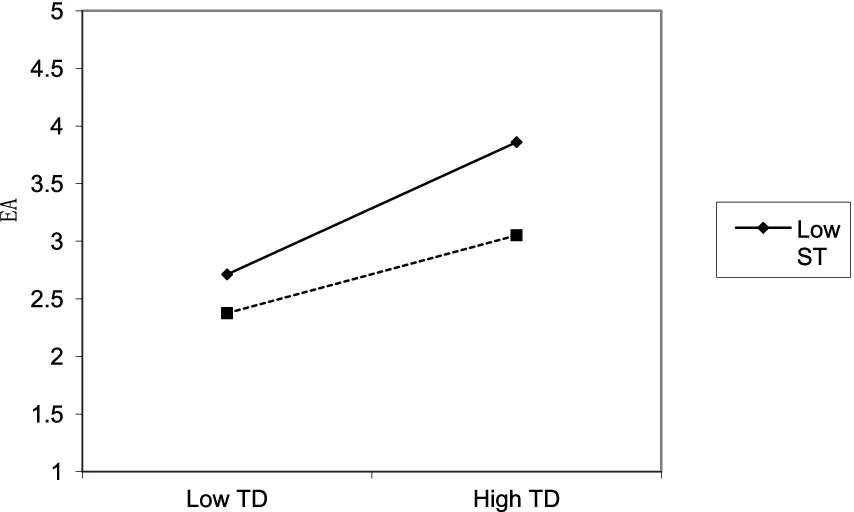
Moderating effect of leader temporal myopia on the relationship between leader-perceived technological dynamism and employee exploratory AI use.

**Figure 4:**
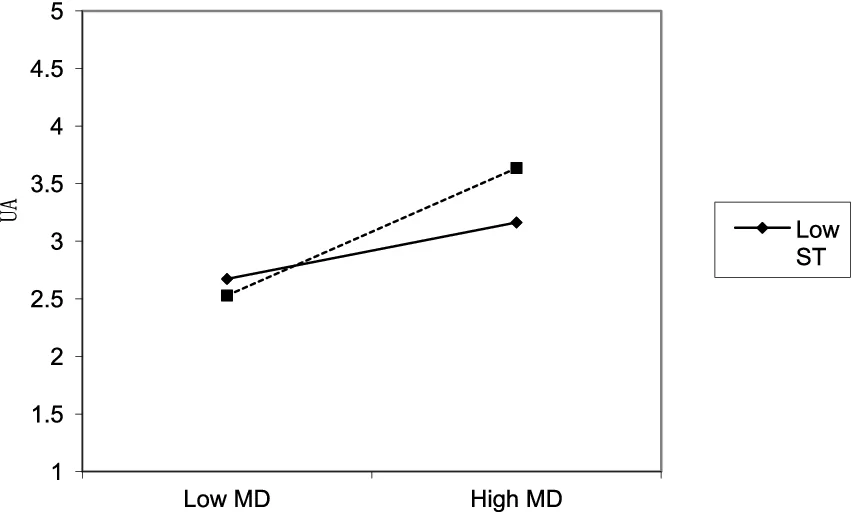
Moderating effect of leader temporal myopia on the relationship between leader-perceived market dynamism and employee exploitative AI use.

Beyond the hypothesized relationships, leaders’ perceptions of technological dynamism were positively related to employee radical creativity (β = 0.132, *p* = 0.035), whereas leaders’ perceptions of market dynamism were negatively related to employee radical creativity (β = −0.170, *p* = 0.001). Regarding the main effects of the moderator, leader temporal myopia was significantly and negatively related to employees’ exploratory use of AI (β = −0.286, *p* < 0.001), whereas its relationship with employees’ exploitative use of AI was not statistically significant (β = 0.082, *p* = 0.102). To further illustrate these moderating effects, we plotted the simple slopes, as shown in Figures 3, 4.

As Figure 3 shows, the positive relationship between leaders’ perceptions of technological dynamism and employees’ exploratory use of AI was stronger when leader temporal myopia was low. When leader temporal myopia was high, this positive relationship became noticeably weaker. This pattern indicates that higher leader temporal myopia attenuates the positive association between perceived technological dynamism and exploratory AI use. Figure 4 shows that the positive relationship between leaders’ perceptions of market dynamism and employees’ exploitative use of AI was stronger when leader temporal myopia was high than when it was low. Thus, higher leader temporal myopia amplifies the positive association between perceived market dynamism and exploitative AI use. Taken together, the simple-slope results provide further support for Hypotheses 3 and 7.

In addition, leaders’ perceptions of technological dynamism had a significant positive indirect effect on employee radical creativity through employees’ exploratory use of AI (β = 0.156, *p* < 0.001, 95% CI [0.099, 0.213]). More importantly, leader temporal myopia significantly attenuated this positive indirect effect (β = −0.040, *p* = 0.021, 95% CI [−0.077, −0.008]), thereby supporting Hypothesis 4.

Leaders’ perceptions of market dynamism also had a significant negative indirect effect on employee radical creativity through employees’ exploitative use of AI (β = −0.095, *p* < 0.001, 95% CI [−0.146, −0.054]). Leader temporal myopia significantly amplified this negative indirect effect—that is, rendered it more negative (β = −0.037, *p* = 0.033, 95% CI [−0.075, −0.008])—thereby supporting Hypothesis 8.

To examine whether the estimated conditional indirect effects were robust to an alternative estimation approach, we conducted supplementary analyses using the PROCESS macro. The PROCESS analyses were based on 10,000 bootstrap samples and 95% confidence intervals and included the same control variables as those used in the PLS-SEM analyses. Because PROCESS estimates the model using composite variable scores rather than latent variables, these results were treated only as supplementary robustness evidence. The primary conclusions regarding the hypotheses therefore remained based on the PLS-SEM latent-variable model. The PROCESS results are reported in Table 6.

**Table 6:** Moderated mediation results.

Path	Moderator	Effect	SE	95% confidence interval
TD → EA → BC	Low ST	0.135	0.037	[0.064, 0.211]
	High ST	0.036	0.019	[0.002, 0.078]
	Difference	−0.099	0.039	[−0.184, −0.033]
MD → UA → BC	Low ST	−0.044	0.021	[−0.087, −0.006]
	High ST	−0.101	0.039	[−0.184, −0.032]
	Difference	−0.057	0.037	[−0.147, −0.003]

β = path coefficient; SE = bootstrap standard error; TD = leader-perceived technological dynamism; MD = leader-perceived market dynamism; ST = leader temporal myopia; EA = exploratory AI use; UA = exploitative AI use; BC = employee radical creativity. f^2^ = effect size; ‘—’ indicates that this statistic is not applicable to an indirect effect.

When leader temporal myopia was low, the indirect effect through exploratory AI use was 0.135 (95% CI [0.064, 0.211]), and the confidence interval excluded zero, indicating a significant indirect effect. When leader temporal myopia was high, the indirect effect was 0.036 (95% CI [0.002, 0.078]), which was also significant. More importantly, the difference between the two conditional indirect effects was significant (high temporal myopia minus low temporal myopia = −0.099, 95% CI [−0.184, −0.033]). These findings indicate a significant moderated mediation effect and provide further support for Hypothesis 4.

When leader temporal myopia was low, the indirect effect through exploitative AI use was −0.044 (95% CI [−0.087, −0.006]), and the confidence interval excluded zero. When leader temporal myopia was high, the indirect effect was −0.101 (95% CI [−0.184, −0.032]), which was also significant. The difference between the two conditional indirect effects was likewise significant (high temporal myopia minus low temporal myopia = −0.057, 95% CI [−0.147, −0.003]). Thus, the negative indirect effect through exploitative AI use became significantly stronger as leader temporal myopia increased, providing further support for Hypothesis 8.

Because the corresponding direct and indirect relationships were both statistically significant and operated in the same direction within each pathway, the findings are consistent with complementary partial mediation rather than full mediation in both the exploratory and exploitative pathways.

## 5 Conclusions and discussion

### 5.1 Conclusion

This study developed a theoretical model linking leaders’ perceptions of environmental dynamism, employees’ AI use modes, leader temporal myopia, and employee radical creativity. It examined how different forms of leader-perceived environmental dynamism correspond to employees’ differentiated AI use behaviors and tested the boundary role of leader temporal myopia. The PLS-SEM results show a significant positive indirect relationship among leader-perceived technological dynamism, employee exploratory AI use, and employee radical creativity; this relationship was weaker when leader temporal myopia was higher. They also show a significant negative indirect relationship among leader-perceived market dynamism, employee exploitative AI use, and employee radical creativity; this relationship was stronger when leader temporal myopia was higher. These results are consistent with the proposed theoretical relationships. However, because the focal variables were not measured at baseline or repeatedly across waves, the findings should be interpreted as temporally separated associational evidence rather than strict causal identification. The results indicate that employees’ use of AI for different knowledge activities is differentially associated with radical creativity and that leaders’ perceptions of environmental dynamism and temporal orientation exhibit distinct patterns of association with employee AI use modes.

### 5.2 Theoretical contributions

First, unlike studies that treat AI use as a unidimensional construct (Davenport and Ronanki, 2018) or distinguish between augmentation and automation according to the division of labor between humans and AI (Raisch and Krakowski, 2021), this study moves beyond the intensity of AI use to examine its functional purposes and knowledge-search orientations. Recent research indicates that AI can perform different functions across idea generation, solution refinement, and implementation, and that its effects depend on the task objectives and knowledge activities it serves (Krakowski, 2025; Gama and Magistretti, 2025; Hou et al., 2025; Huang et al., 2026). Accordingly, we distinguish between exploratory and exploitative AI use based on whether employees use AI to search for distant new knowledge or to refine existing knowledge. This classification concerns employees’ functional deployment of AI in specific R&D activities rather than the exploration–exploitation attributes of the R&D project itself. Our findings demonstrate that these two modes of AI use have distinct relationships with employee radical creativity, thereby providing a more fine-grained explanation for the context-dependent findings concerning the relationship between AI use and employee creativity (Sun et al., 2025; Hou et al., 2025).

Second, although AI can broaden access to knowledge and provide cognitive resources, humans continue to perform critical functions in radical creativity, including problem framing, contextual judgment, and final selection (Ding and Li, 2025; Dell’Acqua et al., 2026). At the same time, employee creativity is not merely the outcome of individual cognition; it is jointly shaped by multilevel conditions involving leaders, organizations, and the external environment (Grilli and Pedota, 2024; Bankins et al., 2024). The adoption and specific uses of AI are similarly embedded in technological, organizational, and external environmental conditions (Gama and Magistretti, 2025; Hughes et al., 2026). Therefore, unlike prior research that has primarily examined AI-related factors as antecedents of creativity, this study incorporates leader-perceived technological and market dynamism into the antecedent framework of AI-use modes. It reveals the distinct relationships between patterns of external environmental change that highlight different knowledge-updating and action-response demands and employees’ exploratory and exploitative AI use. In doing so, this study extends research on the external environmental antecedents of employee radical creativity in the context of AI.

Finally, existing research has begun to examine the outcomes, underlying mechanisms, and individual-level boundary conditions of AI use (Hermann et al., 2025; Sun et al., 2025). However, there remains limited systematic explanation of how leaders shape employees’ different modes of AI use in response to external environmental conditions (Bankins et al., 2024; Hughes et al., 2026). Leaders’ temporal horizons influence the emphasis they place on short-term returns, innovation risks, and delayed benefits, thereby shaping resource allocation for digital and radical innovation (Peng and Yan, 2025; Joo et al., 2025). By jointly examining the antecedents and consequences of different modes of AI use, this study identifies leader temporal myopia as an important boundary condition. Our findings show that leader temporal myopia weakens the relationship between leader-perceived technological dynamism and exploratory AI use while strengthening the relationship between leader-perceived market dynamism and exploitative AI use. In doing so, this study clarifies the relational pathways and boundary conditions linking perceived environmental dynamism, leaders’ temporal orientation, and employees’ modes of AI use.

### 5.3 Practical implications

Because the unit of analysis in this study is the leader–employee dyad, the following managerial implications are derived from findings at the individual and dyadic levels and should not be interpreted as evidence that this study has validated firm-level mechanisms.

First, our findings suggest that organizations can provide the time, resources, and tolerance for failure necessary for exploratory AI use according to the nature of R&D tasks, while avoiding an excessive emphasis on efficiency and short-term delivery that may crowd out distant knowledge search. Exploitative AI use remains appropriate for clearly defined tasks involving data processing, solution screening, and process optimization. However, organizations need to maintain an appropriate balance between efficiency-oriented tasks and highly uncertain exploratory activities. The value of AI should not be understood solely in terms of improving efficiency, reducing costs, and optimizing processes. AI should also be used to extend the boundaries of knowledge search, identify latent relationships, generate new questions, and develop unconventional solutions. In AI-enabled drug discovery in particular, firms should encourage employees to use AI for upstream exploratory activities, such as understanding disease mechanisms, identifying novel targets, integrating cross-domain knowledge, and generating R&D hypotheses, thereby increasing the likelihood that employees will produce highly novel ideas.

Second, organizations should pay close attention to how leaders recognize and interpret environmental dynamism. Our findings indicate that leader-perceived technological dynamism is more closely associated with employees’ exploratory AI use, whereas leader-perceived market dynamism is more closely associated with employees’ exploitative AI use. Organizations should therefore help leaders distinguish between the knowledge-updating and action-response demands highlighted by different forms of environmental change, rather than translating all external changes into pressure for greater short-term speed.

Third, leaders should adopt a long-term orientation toward performance and the value of upstream innovation to prevent temporal myopia from undermining employee radical creativity. Radical creativity is characterized by high uncertainty, long development cycles, and unstable short-term outcomes and therefore requires sustained investment, tolerance for failure, and long-term evaluation mechanisms. When leaders focus excessively on interim performance indicators and immediately visible outcomes, employees may be more likely to use AI to optimize existing tasks and advance current pipelines while reducing their exploration of highly uncertain new problems, mechanisms, and targets. Firms should therefore reduce the weight assigned to isolated short-term indicators in performance evaluations and place greater emphasis on original ideas, the identification of frontier research problems, and high-potential R&D hypotheses.

### 5.4 Limitations and future research

This study has several limitations that should be addressed in future research.

First, although this study draws on the cognitive appraisal perspective to explain why different forms of environmental dynamism may be associated with different behavioral responses, it does not directly measure leaders’ challenge and threat appraisals. We define the two forms of perceived environmental dynamism as leaders’ overall judgments of the rate and unpredictability of environmental change over a given period rather than as cognitive appraisals of a discrete external event. Technological dynamism may simultaneously create new knowledge and innovation opportunities while increasing uncertainty, competitive pressure, and the risk of technological obsolescence. Similarly, market dynamism may involve both response pressures and opportunities arising from new demands. Accordingly, this study does not assign fixed opportunity or threat properties to either form of environmental dynamism. The empirical findings demonstrate only that leader-perceived technological and market dynamism are differentially associated with the two modes of AI use. They do not establish that leaders appraise technological dynamism as a challenge and market dynamism as a threat, nor do they validate these appraisals as mediating mechanisms. Future research could directly measure challenge and threat appraisals and examine how leaders transmit such appraisals to employees through goal setting, resource allocation, and performance signals.

Second, although this study employs a three-wave, multisource leader–employee dyadic design and measures leader-perceived environmental dynamism and temporal myopia, employees’ modes of AI use, and employee radical creativity at different time points in accordance with the proposed theoretical sequence, temporal separation alone is insufficient to establish strict causal relationships. We did not measure employees’ exploratory AI use, exploitative AI use, or radical creativity before the beginning of the study. Consequently, we cannot assess changes in the focal variables relative to their baseline levels or fully rule out reverse causality. For example, employees who already exhibit high radical creativity may be more inclined to use AI in exploratory ways, while employees’ existing modes of AI use may influence leaders’ subsequent evaluations of their creative performance.

Third, teams’ existing project portfolios and R&D activities may also influence leaders’ perceptions of environmental dynamism. For example, teams engaged in frontier technological exploration may make leaders more attentive to technological changes, whereas teams facing commercialization and delivery pressures may heighten leaders’ perceptions of market change. Although this study controls for AI literacy, team size, team age, R&D investment intensity, and project development stage, unobserved factors—including project explorativeness, task novelty, technological uncertainty, and employees’ stable creative dispositions—may simultaneously affect perceived environmental dynamism, modes of AI use, and radical creativity. Moreover, we did not separately measure the exploration–exploitation orientation of the R&D task or project itself. We therefore cannot directly assess the incremental explanatory validity of modes of AI use beyond R&D task orientation within the same model. Future research could measure R&D task characteristics and modes of AI use separately and compare employees’ different AI-use behaviors within the same project or task. Such research would help clarify the empirical boundaries between the two constructs and assess the independent explanatory contribution of modes of AI use.

Fourth, we used a two-week interval between adjacent measurement waves primarily to separate the measurement occasions, maintain relative stability in the project context, and reduce attrition across waves. Because the outcome measured in this study is employees’ creative performance in generating original R&D ideas and novel solutions—rather than final innovation outcomes such as successful drug development or clinical translation—such performance may be observable within a relatively short period. Nevertheless, 2 weeks may not represent the optimal temporal window for examining the relationship between AI use and radical creativity. It is also insufficient to capture the long-term knowledge accumulation, repeated experimentation, and evolution of creativity involved in AI-enabled drug discovery. Future research could repeatedly measure perceived environmental dynamism, modes of AI use, and radical creativity at multiple time points and employ cross-lagged panel models, random-intercept cross-lagged panel models, or latent change models to examine the directional relationships among these variables. Longer observation periods, alternative time intervals, experimental or quasi-experimental designs, records of employees’ prior AI use, and historical creativity evaluations could also help address reverse causality and stable individual differences more effectively.

Finally, this study relies on nonprobability purposive sampling and primarily recruited participants and distributed questionnaires through WeChat, QQ, and email. Firms and respondents willing to participate may have had greater interest in AI and a stronger willingness to cooperate with survey research, meaning that self-selection bias cannot be fully excluded. In addition, we surveyed only one leader–employee dyad from each firm. Although this approach reduces concerns about the non-independence of observations within firms, it cannot capture differences in AI use among employees within the same team or firm and does not permit the identification of team- or firm-level mechanisms. Future research could conduct stratified sampling based on industry directories, collect data from multiple leaders and employees within the same firms and teams, and use multilevel models to distinguish relationships operating at the individual, leader, team, and firm levels.

## Data Availability

The raw data supporting the conclusions of this article will be made available by the authors, without undue reservation.

## Footnotes

1 It is important to clarify that the modes of AI use examined in this study refer to the functional purposes and knowledge-search orientations for which employees employ AI in specific R&D activities, rather than to the overall exploration–exploitation orientation of the R&D projects in which they participate. R&D activities included in the measurement items—such as identifying novel targets, integrating knowledge across domains, screening potential solutions, and optimizing technological trajectories—serve as behavioral criteria for identifying the knowledge-search purposes for which employees use AI; they are not intended to measure the characteristics of the R&D project itself. For example, even in a highly exploratory new drug development project, an employee’s use of AI solely for data processing, candidate compound screening, and parameter optimization would be classified as exploitative AI use. Conversely, in a relatively mature R&D project, an employee’s use of AI to challenge existing assumptions, search for cross-domain knowledge, or propose unconventional solutions would be classified as exploratory AI use. Exploratory or exploitative R&D activities conducted without the use of AI do not constitute exploratory or exploitative AI use as defined in this study.
